# Utilization of Indole Acetic Acid with *Leucadendron rubrum* and *Rhododendron pulchrum* for the Phytoremediation of Heavy Metals in the Artificial Soil Made of Municipal Sewage Sludge

**DOI:** 10.3390/toxics11010043

**Published:** 2022-12-31

**Authors:** Xiaoling Chen, Jianru Feng, Huaqian Mou, Zheng Liang, Tianzheng Ding, Shiyu Chen, Feili Li

**Affiliations:** 1 College of Environment, Zhejiang University of Technology, Hangzhou 310032, China; 2 Jinhua Water Treatment Co., Ltd., Jinhua 321016, China; 3 Shaoxing Institute of Energy Testing, Shaoxing 312000, China

**Keywords:** municipal sewage sludge, phytoremediation, ornamental plant, heavy metal

## Abstract

The development of phytoremediation by garden plants is an effective way to deal with the dilemma of municipal sewage sludge disposal. In this study, two ornamental plants were used as phytoremediation plants to rehabilitate heavy-metal-contaminated municipal sewage sludge in field experiments, and the role of exogenous phytohormone IAA was also tested. Ornamental plants *Loropetalum chinense var. rubrum* (*L. rubrum)* and *Rhododendron pulchrum* (*R. pulchrum*) adapted well to the artificial soil made of municipal sewage sludge, and the concentrations of Cu, Zn, Pb, and Ni were decreased by 7.29, 261, 20.2, and 11.9 mg kg^−1^, respectively, in the soil planted with *L. rubrum*, and 7.60, 308, 50.1, and 17.7 mg kg^−1^, respectively, in the soil planted with *R. pulchrum*, accounted for 11–37% of the total amounts and reached significant levels (*p* < 0.05), except Cd. The concentration of Pb in all parts of the two ornamental plants was increased, as well as most heavy metals in *L. rubrum* root. As a result, three months after transplant, the phyto-extraction amounts in *L. rubrum* were 397, 10.9, and 1330 μg for Ni, Cd, and Pb, respectively, increased by 233% to 279%. The phyto-extraction amount in *R. pulchrum* were 1510, 250, and 237 μg for Zn, Pb, and Cu, respectively, increased by 143% to 193%. These results indicated a potential to remediate heavy metals of the two ornamental plants, especially *L. rubrum*. The results of correlation analysis implied that the interaction of heavy metals in the plant itself played an important role in the uptake of heavy metals. This seemed to explain why applying IAA in the experiment had little effect on plant growth and phytoremediation of heavy metals. This study provided a green and feasible idea for the proper disposal of municipal sewage sludge.

## 1. Introduction

The rapid rise in industrialization and urbanization has enhanced the production of sewage. Therefore, a large amount of sewage sludge has been produced after wastewater treatment, and it is speculated to increase further in the near future [1,2]. In 2019, China’s annual municipal wastewater treatment capacity reached 52.5 billion m^3^, generating 11 million tons of dry sewage sludge [3]. It was documented that approximately 10 million dry tons of sewage sludge were produced annually in EU countries [4] and about 45 million dry tons globally [5]. More than 80% of sewage sludge was improperly disposed of, which may cause serious secondary pollution, such as heavy metals, to the environment [2,6]. Heavy metals in sewage sludge mainly come from agricultural, industrial, and traffic activities [7,8]. Therefore, speeding up sewage sludge treatment and disposal is imminent.

Compared with waste landfill and other disposal methods, sewage sludge land use is economically feasible and environmentally sustainable [9]. Moreover, the use of sewage sludge as fertilizer can provide nutrients to plants and improve the properties of the soil [10,11], reduce the need for synthetic inorganic fertilizers, and save non-renewable energy [12]. However, heavy metals contained in sewage sludge cannot be biodegraded and have attracted more and more attention due to their potentially dangerous, chronic, and irreversible characteristics [13]. For example, copper is an essential trace element for human health, but Cu exposure produced a high potential non-carcinogenic risk to human health, especially children [14]. In addition, exposure to Cd and Pb could cause itai-itai disease, blood poisoning, and anemia [15]. Heavy metals also cause adverse effects on crop growth and pollute groundwater, soil, and the food chain [7,16]. Therefore, sewage sludge must be properly treated before being applied to soils. The most common methods include anaerobic digestion, aerobic composting, lime stabilization, incineration, and pyrolysis. These methods aim to stabilize the sewage sludge, eliminate its potential environmental pollution and restore its agronomic value [17]. Repairing these heavy metal contaminations is of great significance to maintaining environmental stability [18,19].

On the other hand, heavy-metal-contaminated soil can be repaired by a variety of physical, chemical, and biological techniques [20,21]. However, physical/chemical methods are faced with high cost, low energy efficiency, interference with soil properties, secondary pollution, and other problems [22]. In contrast, phytoremediation has been considered to be a cost-effective and environmentally friendly remediation mechanism for remediating metal-contaminated soil [23]. Hyperaccumulators can accumulate a large number of heavy metals in shoots, but their remediation efficiency is limited due to their small biomass and slow growth rate [24,25].

As a low carbon emission process, the combined technology of aerobic digestion and garden land use has been recommended by many governments as the preferred technology for sewage sludge treatment and disposal. This coupled process can not only promote soil organic carbon accumulation but also reduce greenhouse gas emissions during fertilizer production by replacing chemical fertilizers, which is conducive to achieving the objectives for carbon dioxide peaking and carbon neutrality. The innovation of this study lies in the selection of suitable landscape plants and the combination of artificial regulation technology to achieve the reduction of heavy metals in soil, and new evaluation factors for phytoremediation plants were proposed.

Some garden plants have a strong tolerance to heavy metals and have the characteristics of large biomass and a wide cultivation range [26]. However, the remediation is also dependent on the composition of soil and plant microbe interaction [27], added nutrient and amendment [28], and the exogenous plant growth regulators [29]. Under heavy metal stress, the decrease in plant growth and biomass resulted in a decrease in bioremediation efficiency [30]. In order to improve the uptake and accumulation of metals in plants, various strategies have been used, including fertilizers, chelating agents, surfactants, and plant growth regulators (PGRs) [31,32,33,34]. For example, a study has shown that exogenous indoleacetic acid (IAA) can alleviate the stress of heavy metals on plants by changing plant growth parameters [31]. Therefore, there are still many ornamental plants that need to be evaluated for their ability to extract heavy metals from sewage sludge.

*Loropetalum chinensevar rubrum* (*L. rubrum*) and *Rhododendron pulchrum* (*R. pulchrum*) are two common ornamental shrubs and showed relatively high transport factors (TF) for heavy metals in our preliminary investigation. Field experiments were conducted to investigate the effects of exogenous IAA application on the heavy metal accumulation of the two species grown in contaminated soil. In the experiment, plants were grown in soil contaminated by heavy metals (including Cu, Zn, Pb, Cd, and Ni). 

The main objectives of this work were (1) To evaluate the remediation potential of ornamental plants to heavy metals in the artificial soil prepared from composted sewage sludge; (2) To evaluate the effects of exogenous IAA on the accumulation and transport of heavy metals in different organs of plants growing in heavy metal contaminated soil.

## 2. Materials and Methods

### 2.1. Experimental Site and Characterization of Soils

The site of this experiment is located in Sanjiangkou Park (29.91° N, 121.54° E), Jiangbei District, Ningbo City, Zhejiang Province, P. R. China. It belongs to the subtropical monsoon climate, specified by a warm and humid climate.

The tested artificial soil for the landscaping substrate was provided by Ningbo High-tech Zone Chunli Energy Saving Technology Co., Ltd., Ningbo, China. The artificial soil is composed of municipal sewage sludge after aerobic compost as the main material (accounting for about 60% of the total), and rice husk, lime, biochar, and clay as the auxiliary materials to form a dark brown soil, named nutrient soil. The nutrient soil was approximately 0.98 g cm^−3^ in bulk density, 8.0 in pH, 65 g kg^−1^ in organic matter, and 3% in total nutrients (Table 1). Artificial soil with slightly alkaline and high organic matter is beneficial to plant growth and soil conditioning [35]. 

### 2.2. Experimental Design

In this experiment, *L. rubrum* and *R. pulchrum* were selected from the plants newly planted in the park. The planting density was 9 plants m^−2^. IAA (indole-3-acetic acid, CAS 87-51-4) was purchased online and applied according to the following experimental design.

**Figure 1:**
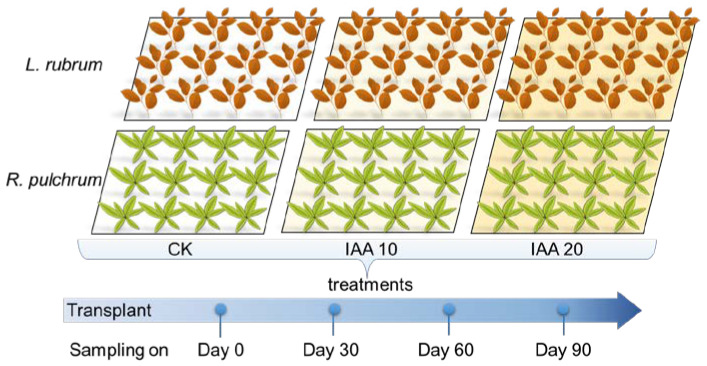
The schematic of experiment design and sampling.

The initial properties of plants and soil were measured before plants were transplanted on artificial soil and defined as the pre-treatment samples (named PT, Figure 1). Then, the area of each plant was divided into three blocks, corresponding to three different treatments. IAA was sprayed as the plant growth regulator on the leaves of each plant. The reported dose of IAA ranged from 4.4 to 100 mg L^−1^ [33,34,36], with effects being species-specific. Therefore, the commonly used concentrations of 10 mg L^–1^ and 20 mg L^–1^ were selected to examine the effects on the phytoremediation efficiency of the two plants. The left block was sprayed with the sterilized deionized water, defined as the control group (named CK). Plants and soils of the experimental groups and control groups were sampled on days 30, 60, and 90 after transportation and were put together to represent the average effect of each treatment over the three months. All samples were randomly collected, and a consistent pretreatment process was used. 

### 2.3. Sample Analysis

Plant height, biomass, and chlorophyll were determined with fresh plants. Three plants were randomly selected under each treatment, and the plant height was immediately determined by measuring the height from the top to the mulch with a ruler. Then the roots, stems, and leaves were separated, washed with tap water, and then rinsed gently with deionized water three times, then blotted up the moisture with paper. The biomass was measured immediately by a balance. Then, 1 cm^2^ fresh leaf was cut into filaments about 5 mm wide and 1 mm wide and dissolved with 80% aqueous acetone solution overnight; then the absorbance at 663 nm and 645 nm was measured with an ultraviolet spectrophotometer (Shunyu 756PC, Shanghai, China). The chlorophyll content was calculated as follows [37].

Chl_a_ = 12.7 A_663_ − 2.69 A_645_ (1)

Chl_b_ = 22.9 A_645_ − 4.86 A_663_ (2)

*C*_A_ = (Chl_a_ + Chl_b_)/2 (3)

A_663_ and A_645_ are the absorbances at 663 and 645 nm, respectively; Chl_a_ and Chl_b_ are the content of chlorophyll a and b, respectively, μg mL^–1^; *C*_A_ is the content of chlorophyll per unit area, mg dm^–1^.

The soils were air-dried, removed from debris and ground, and passed through a 20-mesh sieve. Under the condition of a water-soil ratio of 2.5:1 (w:w), the soil pH was measured with a pH meter (Leici phS-3C, Shanghai, China) [29]. The soil organic matter (SOM) was determined by adding concentrated sulfuric acid to the potassium dichromate solution and oxidizing the soil at 170~180 °C [38]. Cation exchange capacity (CEC) was determined with barium chloride buffer solution (Environmental Standard of China, HJ 889–2017).

A part of the plant and soil samples were air-dried and ground to pass through a 100-mesh sieve for the analysis of heavy metal concentrations. About 0.35 g plant sample or 0.05 g soil sample was digested by a microwave digestion instrument using HNO_3_-HF-H_2_O_2_ (6:2:1, v:v:v) or HNO_3_-HF-H_2_O_2_ (8:1:1, v:v:v), respectively. After the digestion solution was diluted with 5% HNO_3_ to a constant volume, the heavy metal concentrations were determined by flame atomic absorption spectrometry (AAS, PE AAnalyst 800, America) [39].

### 2.4. Evaluation of the Transportation Ability of Heavy Metals

The bioconcentration factor (BCF) and transport factor (TF) of a plant [37], as well as the phytoextraction amount (PEA), were used to evaluate the ability of plants to accumulate, translocate and remove heavy metals. The calculation formulas are as follows.

BCF = *C*_root_/*C*_soil_ (4)

TF = *C*_above_/*C*_root_ (5)

*C*_above_ = (*C*_leaf_ × *M*_leaf_ + *C*_stem_ × *M*_stem_)/(*M*_leaf_ + *M*_stem_) (6)

PEA = *C*_leaf_ × *M*_leaf_ + *C*_stem_ × *M*_stem_ (7)

where *C*_root_, *C*_soil_, *C*_above_, *C*_leaf_, and *C*_stem_ are the concentrations of heavy metal (mg kg^−1^) in the root, soil, and the aboveground part (leaf + stem), leaf, and stem, respectively; *M*_leaf_ and *M*_stem_ represent the weight (g) of the plant leaf and stem, respectively; PEA is the phyto-extraction amount, μg plant^−1^.

### 2.5. Statistical Analysis

All data are analyzed by the software SPSS 19.0 statistically. The test results of normal distribution showed that the kurtosis and skewness of all data varied between −2.1 and 6.9 and between −1.3 and 2.1, less than 10 and 3, respectively, and a normal distribution could be basically accepted. For the one-way analysis of variance (ANOVA) test, the Tamhane method was used. The difference was considered statistically significant when *p* < 0.05. All graphics were drawn using the software Origin 9.0.

The reagents used in the experiments were of analytical purity or higher. Solutions were prepared and diluted with milli-Q water (Millipore, Burlington, MA, USA). All glassware used was soaked in 10% (*v/v*) HNO_3_ solution for more than 24 h and then rinsed three times with tap water and finally with milli-Q water. 

The standard references of soil (GBW07429) and tea (GBW07605) approved by the National Research Center for certified reference materials (Beijing, China) were used for accuracy quality control, and the relative standard deviation of five heavy metals were less than 5%. The standard solution of each heavy metal (1000 mg L^−1^) obtained from the National Institute of Metrology, China, was diluted to appropriate concentrations with 2% (*v/v*) HNO_3_ for calibration of AAS.

## 3. Results and Discussion

### 3.1. Garden Plants Growth Situation

**Figure 2:**
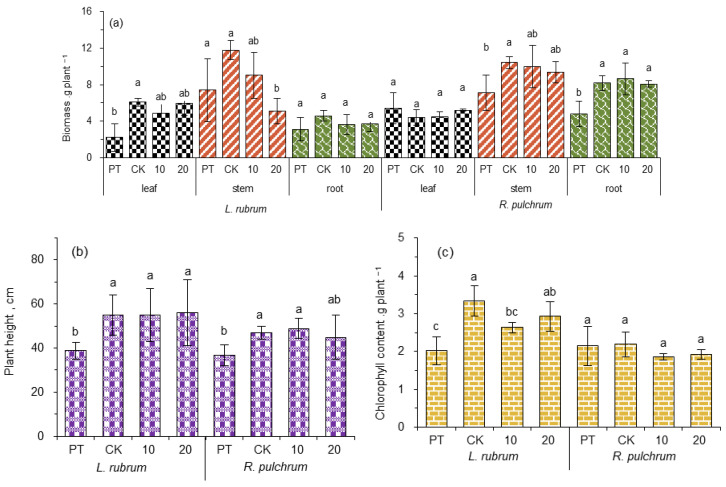
Influence of exogenous IAA treatment on (**a**) biomass, (**b**) plant height, and (**c**) chlorophyll content of *L. rubrum* and *R. pulchrum*. Different letters in the same column of the same plant indicate significant differences among the treatments, *p* < 0.05. The posthoc test was performed with Tamhane method. PT indicates samples of pre-treatment, while CK, 10, and 20 are IAA treatments at 0, 10, and 20 mg L^−1^, respectively.

Usually, accelerating the growth rate or increasing the biomass of plants are effective ways to improve the efficiency of phytoextraction to heavy metals. IAA is a plant growth hormone that is beneficial to both root and shoot development [40], and this is why IAA was chosen to conduct this study. In order to evaluate the potential of *L. rubrum* and *R. pulchrum* to repair heavy metals, the plant growth characteristics under IAA treatments were compared by measuring plant height, biomass, and chlorophyll content (Figure 2).

Compared with PT, the biomass and plant height of the two plants of CK were significantly increased (*p* < 0.05), except for the biomass of the root of *L. rubrum* and leaf of *R. pulchrum*. The results suggest that these two kinds of ornamental plants grew well after transplantation, which indicated that they could survive in the high concentrations of heavy metals contained in the artificial soil. This tolerance to heavy metal stress makes them promising as remediation plants for sewage sludge land use. In addition, the chlorophyll content of *L. rubrum* in CK increased significantly (*p* < 0.05), which might be attributed to the sensitivity of *L. rubrum* to environmental factors. It was reported that with the increase in temperature and light intensity, the rate of chlorophyll synthesis in leaves increased [41]. During the observation period from March to June, the study site changed from spring to summer, and the temperature gradually rose.

However, as compared to the CK, instead of promoting the growth of the plants, the application of 20 ppm IAA decreased the stem biomass of *L. rubrum* by 56.8% (*p* < 0.05), with other indicators not changing at significant levels. However, this result was unexpected. It was probably because that the used dose levels were not sufficient to induce growth promotion in the open field experiment. Usually, the effect of a plant growth regulator is related to its dose and plant species [31]. Yuqin Liang et al. found in a greenhouse experiment that there was no significant change in the shoot biomass after spraying IAA at concentrations of 10 and 50 μmol·L^−1^ (1.7 and 8.7 ppm) on the leaves of *Sedum alfredii* Hance (*S. alfredii*) [40]. Therefore, it was deduced that larger doses of IAA were required in such a field experiment, probably due to the fast dissipation of sprayed IAA on leaves to the surrounding environment.

### 3.2. Changes in Physicochemical Properties of Soil

**Figure 3:**
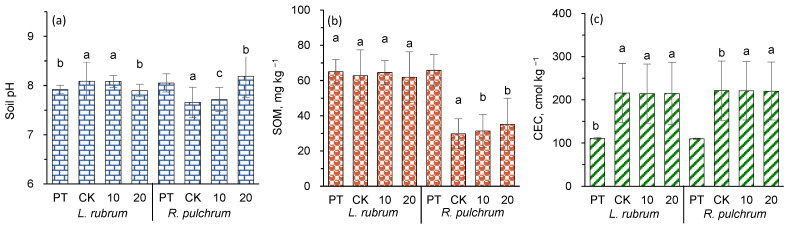
Effects of planting ornamental plants and applying IAA on (**a**) soil pH, (**b**) SOM, and (**c**) soil CEC. Different letters indicate significant differences among the treatments, *p* < 0.05. The posthoc test was performed with Tamhane method. PT indicates samples of pre-treatment, while CK, 10, and 20 are IAA treatments at 0, 10, and 20 mg L^−1^, respectively.

Figure 3 shows the physicochemical properties of soil treated with various doses of IAA, before (PT) and after *L. rubrum* and *R. pulchrum* were transplanted. The soil’s physical and chemical properties include soil pH, SOM, and CEC. 

Compared to PT, the average soil pH in CK was significantly decreased by 0.40 units in soil planted with *R. pulchrum* but increased by 0.17 (*p* < 0.05) in soil planted with *L. rubrum*. The research of ten urban greening tree species showed that *Rhododendron simsii* had the highest number of compounds, with 89 species in root exudates, while the *Loropetalum chinense var. rubrum* had 57 species [42]. The carbon exudation of fine and coarse roots of *Rhododendron lauranum* was up to 18.22 to 10.47 μg C g^−1^ d.wt.hr^−1^, with both organic acid and amino acid accounting for ~ 36% of the total extracts of *Rhododendron groenlandicum* [43]. These cases supported the deduction that *R. pulchrum,* in the same genus as *R. lauranum*, produced a lot of root exudates which facilitated microbial growth, while the organic acids in root exudates reduced the soil pH. The increase in pH in soil planted with *L. rubrum* could be attributed to the decarboxylation of organic anions in root exudates during plant growth, as reported in an earlier study [44]. 

In comparison to PT, the concentration of SOM in the soil grown by *R. pulchrum* was decreased by 54.8% (*p* < 0.05), while the concentration of SOM in soil grown by *L. rubrum* did not change. The CEC concentrations were significantly increased (*p <* 0.05) in both soils grown by *L. rubrum* and *R. pulchrum*. The changes in soil physicochemical properties could be attributed to the different rhizosphere effects in different plants. It was reported that rhizosphere effects differ according to plant species due to differences in the nature of their exudates, nutrient acquisition strategies, and root system architecture [45]. In addition, during the observation period from March to June was a transition from spring to summer, and the temperature rose. The high temperature was conducive to the rapid decomposition of SOM [10] and the humification of biological residues in the soil [46], and the latter may be the cause of the increase in CEC. However, *L. rubrum* had less root biomass than *R. pulchrum* (Figure 2a) and, as a result, a weaker rhizosphere effect. An interesting phenomenon was that SOM in *R. pulchrum* plante soil was much higher than that in *L. rubrum* planted soil. It was deduced that the huge root system of *R. pulchrum* was conducive to producing a high level of biological activity of the root microbiome. Some previous studies reported that roots promote microbial growth and enzyme production, which accelerated the degradation of SOM [47].

The effects of IAA foliar spraying on soil’s physical and chemical properties should be indirect and related to the plant itself. In our research, only the dose of 20 ppm IAA had significantly increased pH in soil grown by *L. rubrum* (*p* < 0.05), compared with CK. The SOM and CEC in the two plant soils did not show significant changes with the application or increment of IAA. It was found that exogenous IAA increased the rhizosphere pH of wheat under Al stress [36], but when exogenous IAA was used to assist *Amaranthus hypochondriacus* L. remediating Cd contaminated soil, soil pH decreased significantly [29]. 

### 3.3. Heavy Metal Concentrations in Soil

**Figure 4:**
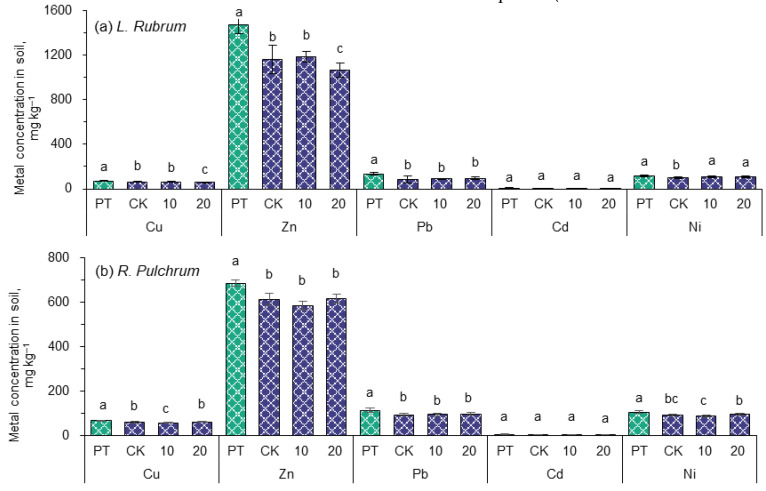
The effects of planting garden plants (**a**) *L. rubrum*, (**b**) *R. pulchrum* and applying IAA on residual concentrations of heavy metals in soil. The soil residual concentrations of heavy metals after planting garden plants and applying IAA. Different letters indicate significant differences among the treatments, *p* < 0.05. The posthoc test was performed with Tamhane method. PT indicates samples of pre-treatment, while CK, 10, and 20 are IAA treatments at 0, 10, and 20 mg L^−1^, respectively.

The concentrations of heavy metals in the soil were measured and expressed as an average of each treatment over a three-month observation period, as shown in Figure 4. The concentrations of Cu, Zn, Pb, Cd, and Ni in the PT group were consistent with their initial concentrations in the artificial soil, listed in Table 1. After the garden plants were grown, the concentrations of the above metals in CK were in the range of 61.3–63.4 mg kg^−1^, 1090–1160 mg kg^−1^, 83.8–94.6 mg kg^−1^, 4.62–5.42 mg kg^−1^, and 93.4–98.1 mg kg^−1^, respectively. These concentrations were decreased by 7.3 to 310 mg kg^−1^ compared with PT, reaching significant levels (*p <* 0.05), except for Cd. The decrease in heavy metal concentrations in the artificial soils was in line with the experimental expectations, which should thank the extraction of the two ornamental plants (discussed in the next section). 

After spraying 10 ppm IAA on plant leaves, the concentrations of Cu in the soil planted with *R. pulchrum* decreased significantly compared with CK, while the other metals were changed little. The situation in the soils planted with *L. rubrum* was a bit different. The concentrations of all heavy metals had no significant difference compared to the CK, except Ni increased significantly (*p* < 0.05). However, increasing the dose of IAA to 20 ppm, the concentrations of Cu and Ni in the soil of *R. pulchrum* increased by 5.3% and 9.2% (*p* < 0.05), respectively, compared to the dose of 10 ppm IAA, but there was no difference compared to the control group. However, in the soil of *L. rubrum* planted, the concentrations of Cu and Zn were decreased by 6.1% (*p* < 0.05) and 10% (*p* < 0.05), respectively. Overall, under the application of IAA, only the removal of Cu and Zn in the soil planted by *L. rubrum* and Cu in soil planted by *R. pulchrum* were promoted, and the effects depended on the dose of IAA. Whether it could be attributed to the promotion of IAA and its mechanism of action, need to be further studied.

### 3.4. Heavy Metal Concentrations in the Ornamental Plants

**Figure 5:**
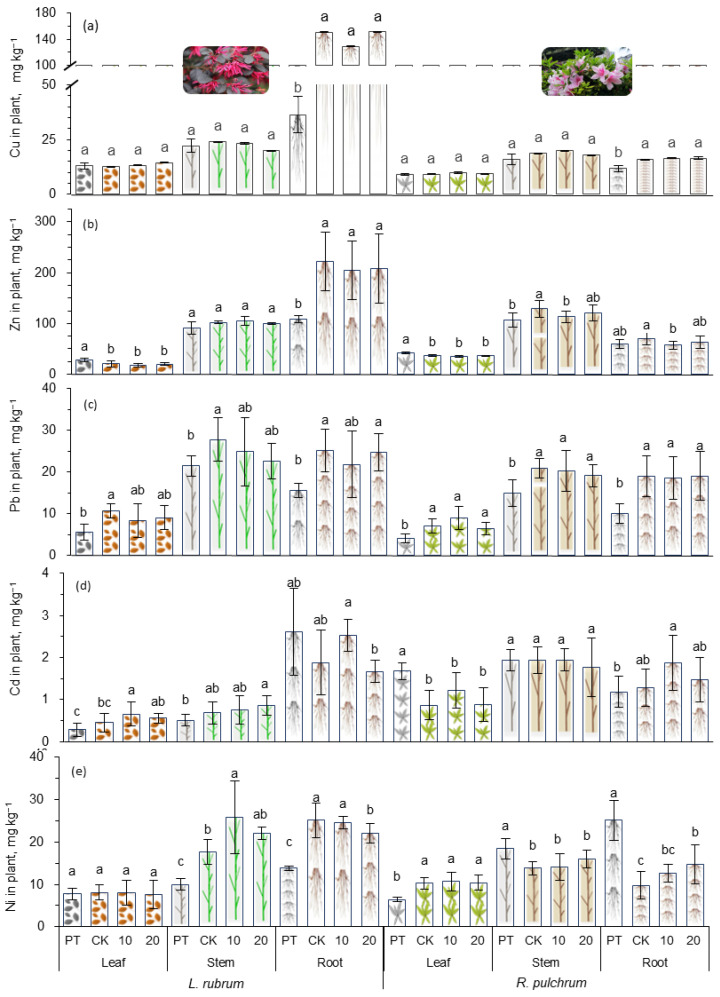
Heavy metal concentration in different parts of the ornamental plants under heavy metal stress and applying IAA. In the figure (**a**–**e**) are the effects of Cu, Zn, Pb, Cd and Ni on concentrations in different plant parts. Different letters indicate significant differences among the treatments, *p* < 0.05. The posthoc test was performed with Tamhane method. PT indicates samples of pre-treatment, while CK, 10, and 20 are IAA treatments at 0, 10, and 20 mg L^−1^, respectively.

The concentrations of heavy metals (mg kg^−1^, dry mass) in different parts of the plants are shown in Figure 5. Compared with the PT group, the variation rule of the heavy metals in *R. pulchrum* of CK was as follows, the concentration of Pb in leaves, stems, and roots increased by 40% to 89% (*p* < 0.05), while that of Cu changed insignificantly, and the concentrations of other metals increased or decreased occasionally. The variation rule in *L. rubrum* was slightly different; the concentration of Pb in all parts was increased significantly by 30% to 93% (*p* < 0.05), and all metals except Cd in roots were increased significantly (*p* < 0.05). The increase in heavy metal concentration in plants should be related to the high concentrations of heavy metals in the artificial soils, demonstrating the potential of these two plants to remediate heavy metals derived from sewage sludge. It has been reported that the concentration of Zn, Pb, and Cd in plants can be up to 1120 mg kg^−1^, 370 mg kg^−1^, and 20.5 mg kg^−1^, respectively, when grown in heavily polluted soil derived from mining areas [48]. Zeng et al. suggested that *L. rubrum* is a potential candidate for phytostabilization in cadmium-contaminated soils [49]. Zu Yanqun et al. found that *Rhododendron annae* can be used as a candidate plant for the accumulation of heavy metals [48]. In addition, the bioaccumulation of Cd was low and changed insignificantly after the two plants were planted in artificial soil, which may be due to its inactivity at the high pH of the artificial soil and adsorption by clay. It has been documented that plants had higher Cd bioaccumulation under weak acid conditions [50]. Additionally, the adsorption of clay minerals added during the production of the artificial soil should also be considered [51].

In general, the effect of IAA application on the phytoextraction efficiency of heavy metals varied with plants, heavy metal elements, and applied doses. In the study, the addition of IAA did not change the concentrations of heavy metals in both plants, except that it significantly increased Ni in *R. pulchrum* root under the dose of 20 ppm (*p* < 0.05) and Cd in *L. rubrum* root under the dose of 10 ppm. This was consistent with the result of Chen’s research that the application of 1 μM IAA in *Sedum alfredii* had no significant effect on heavy metal content in plants [52]. However, our results also seemed to be contrary to some existing reports. For example, a study showed that the Pb concentration in maize shoots decreased significantly with the addition of IAA due to the stronger competition of Ca for binding the sites on the transporting transmembrane protein [33]. Compared with the control group, a pot experiment with a concentration of Cd of 100 mg kg^−1^ and sprayed with 10 μM IAA every 10 days showed that the concentration of Cd in roots and leaves of *Dysphania ambrosioides* was increased by 178% and 118%, respectively [37]. After applying 500 mg L^−1^ IAA, the Cd concentration in the shoots of *Helianthus annuus* L. increased from 17.41 to 43.89 mg kg ^−1^ in the polluted soil of 15 mg Cd kg^−1^ [53], which was approximately three times the concentration of Cd in this research. It is easy to see that the above findings were derived from different plant species, different doses of IAA, and different levels of pollution. Therefore, the effects of exogenous phytoregulators on the phytoremediation of plants to extract heavy metals are extremely complex, and there may be many more extensive studies needed to eliminate differences and grasp the rules. 

### 3.5. Bioconcentration and Transport Factors of Heavy Metals by Ornamental Plants

**Figure 6:**
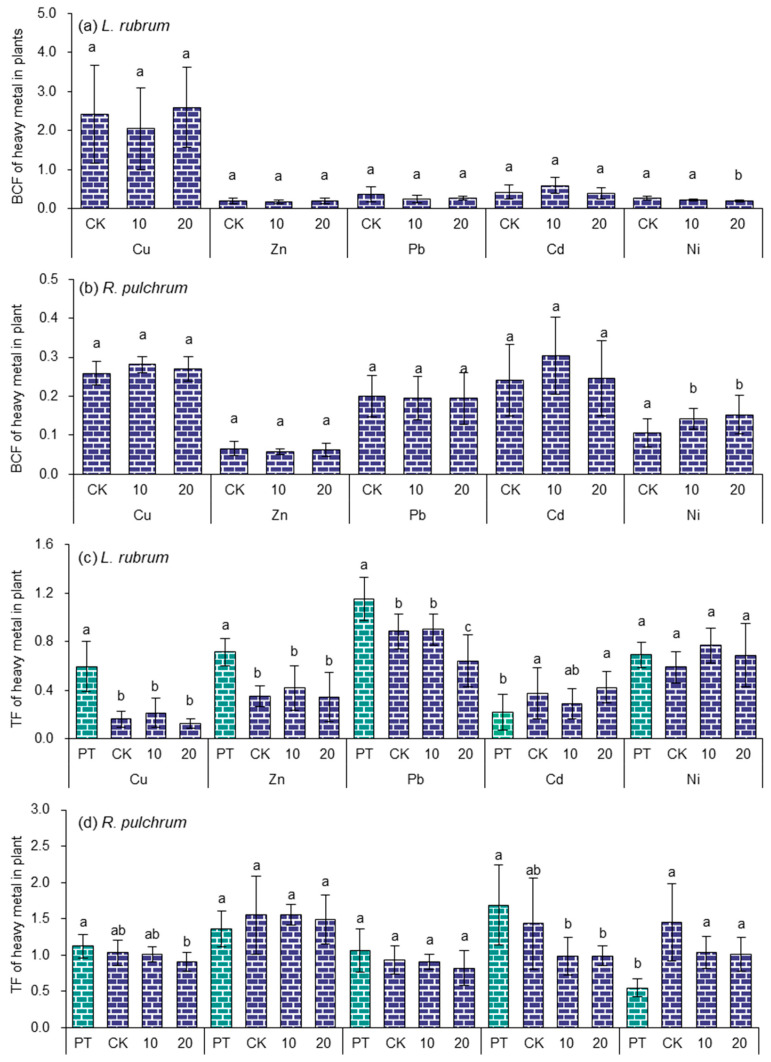
Effects of heavy metal stress and IAA application on BCF and TF values of ornamental plants, (**a**,**b**) are BCF values of *L. rubrum* and *R. pulchrum*; (**c**,**d**) are TF values of *L. rubrum* and *R. pulchrum*, respectively. Different letters indicate significant differences among the treatments, *p* < 0.05. The posthoc test was performed with Tamhane method. PT indicates samples of pre-treatment, while CK, 10, and 20 are IAA treatments at 0, 10, and 20 mg L^−1^, respectively.

The bioconcentration factor (BCF), and transport factor (TF), which can exclude the heavy metal concentration differences in soil or root of plants [54,55], were used to evaluate the accumulation and transportation characteristics of heavy metals in soil-ornamental plant system, respectively, as shown in Figure 6.

Since the ornamental plants were all obtained from the market, the original soil information was lost, and their initial BCF values could not be calculated. Only the BCF values of the two ornamental plants to heavy metals after transplantation were discussed. In the CK group, where ornamental plants were transplanted to the artificial soil, the BCF values of heavy metals of *L. rubrum* and *R. pultrum* ranged from 0.20 to 2.41 and from 0.066 to 0.26, respectively. In general, the BCF values of most plants were less than 1. For instance, according to Urszula Wydro’s research, after one year of mixed seeding of *Lolium perenne*, *Poa pratensis*, and *Festuca rubra* in sludge-applied soil, the BCF values of Cu, Zn, Pb, and Ni were all less than 0.8, and the BCF value of Pb was as low as 0.02 [56]. Another reason for the lower *BCF* values could be due to the higher concentration of heavy metals in the artificial soil. Chen et al. found that when *Sedum alfredii* was planted in slightly heavy-metal-contaminated soil, the BCF was much higher than that in heavily heavy-metal-contaminated soil [57]. One interesting thing in the study was that the BCF value of Cu in *L. rubrum* was larger than 1, and the BCF values of other heavy metals were larger than those of *R. pulchrum*, indicating a great potential to remediate heavy metals in the sewage sludge. *L. rubrum* had been reported as a remediation plant for heavy metals by Chen [53]. 

However, the application of IAA played little effect on the BCF of all heavy metals of the two ornamental plants, except that it increased the BCF of Ni in *R. pultrum* (at the dose of 10 and 20 ppm) and decreased the BCF of Ni in *L. rubrum* (at a dose of 20 ppm). This result could be attributed to the limited effect of the exogenous application of plant growth regulators on the accumulation of heavy metals in plant roots. A study also showed that BCF and TF of Zn in *S. alfredii* were not significantly affected by the application of 0.2 mg L^−1^ [57].

The TF values of plants to different heavy metals vary greatly. In the CK group, TF values of heavy metals in *R. pulchrum* were in the order of Zn (1.55) > Ni (1.45) > Cd (0.43) > Cu (1.03) > Pb (0.93), and the TF of Ni was 2.6 times that of the CK (*p* < 0.05). The order in *L. rubrum* was Pb (0.89) > Ni (0.59) > Cd (0.38) > Zn (0.35) > Cu (0.16), and the TF of Cu, Zn, and Pb were 0.27-, 0.50-, 0.77-fold that of CK (*p* < 0.05), respectively. Most of the TF values in *R. pulchrum* were greater than 1, suggesting that it is a potential remediation plant with good heavy metal migration ability. It was also close to the result of the research with *Azolla caroliniana*, which was reported as a potential accumulator for heavy metals, that it had higher bioconcentration factors of 0.37–1.4 for various heavy metals [58]. In addition, the TF values of different heavy metals vary greatly among different plants. Some hyperaccumulator, such as *S. alfredii* has TF value as low as 0.085 for Pb [57], which was even lower than those of heavy metals in *L. rubrum*. 

After applying IAA, there was no significant difference in TF values in plants among treatments, except TF of Pb in *L. rubrum* at the dose of 20 ppm. Previous studies have shown that exogenous application of IAA can reduce the stress of heavy metals on different plants, promote plant growth, improve the absorption and transport of heavy metals, and improve the repair efficiency of heavy metals [34,59,60]. However, there are also reports on the contrary. Zhiqin Chen et al. sprayed IAA to *S. alfredii* planted in the soil polluted by heavy metals but found that the TF value of Zn had no change [57]. Ji et al. found applying IAA did not affect the Cd translocation factor for *Solanum nigrum* [34]. In addition, Fassler pointed out that the effect of IAA in reducing heavy metal stress to plants would fail if the concentrations of heavy metals in soils were too high [59]. 

It follows that spraying exogenous phytoregulators had a limited effect on the enrichment and transport of heavy metals in garden plants. The reason could be either the doses of IAA that did not match environmental conditions, such as plant species, the heavy metals, and their concentrations in soil, or the concentrations of heavy metals in soil were too high to be regulated by the IAA. 

### 3.6. Phytoremediation of Heavy Metals by Ornamental Plants

**Figure 7:**
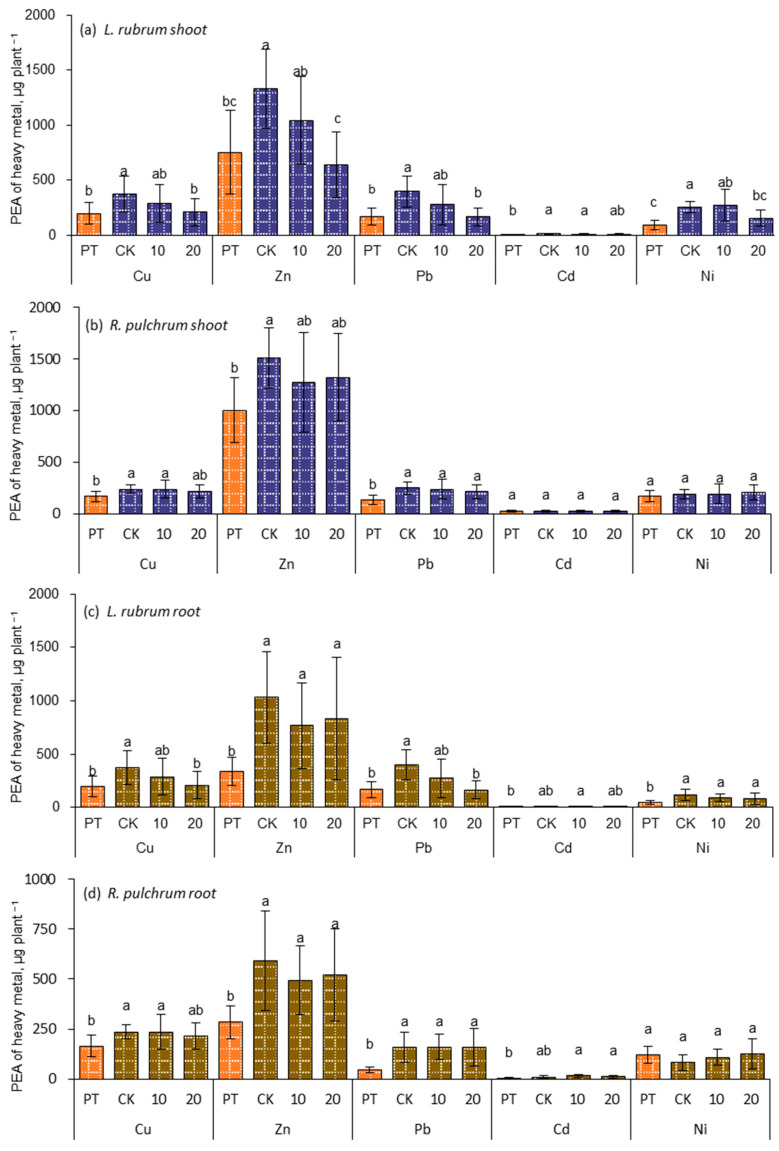
Heavy metal accumulated in the shoot and root of the garden plants, (**a**,**b**) are the plant extraction amount of heavy metals in the shoot of *L. rubrum* and *R. pulchrum*, and (**c**,**d**) are the plant extraction amount of heavy metals in the roots of *L. rubrum* and *R. pulchrum*. Different letters indicate significant differences among the treatments, *p* < 0.05. The posthoc test was performed with Tamhane method. PT indicates samples of pre-treatment, while CK, 10, and 20 are IAA treatments at 0, 10, and 20 mg L^−1^, respectively.

The main reason for choosing ornamental plants as remediation plants is their larger biomass than those hyperaccumulators and their strong adaptability [61]. Therefore, the phyto-extraction amount (PEA) of heavy metals by plants, expressed by the total accumulated heavy metal in shoots (Equation (7)), was used to assess the ability of the plants to remove metals, as seen in Figure 7. 

The aboveground biomass of both plants was larger than that of the root, so the PEA value of the aboveground part was larger than that of the root in most cases. In addition, garden plants have to be pruned at least twice a year due to the requirements of management and aesthetics, which further increases the significance of the extraction amount of the aboveground part. Therefore, the PEA of heavy metals from the shoots and roots were calculated, respectively. For PEA of plant shoot, heavy metals in per *R. pulchrum* followed the trend of Zn (1510 μg) > Pb (250 μg) > Cu (237 μg) > Ni (191 μg) > Cd (23.9 μg). Thereinto, Zn, Pb, and Cu were 150%, 193%, and 143% greater than that in the PT, respectively, and reached a significant level (*p* < 0.05, Figure 7a). The PEA in per *L. rubrum* were sorted in the following order: Zn (1330 μg) > Pb (397 μg) > Cu (372 μg) > Ni (253 μg) > Cd (10.9 μg), and were significantly greater (*p* < 0.05) than the corresponding PEA in the PT by 177%, 235%, 191%, 279%, and 233%, respectively, (Figure 7b). For plant root, the PEA in per *R. pulchrum* followed the trend of Zn (592 μg) > Cu (237 μg) > Pb (161 μg) > Ni (83 μg) > Cd (11 μg). Thereinto, Pb, Zn, and C were 344%, 208%, and 197% greater than that in the PT, respectively, and reached a significant level (*p* < 0.05, Figure 7c). The PEA by root in per *L. rubrum* were ordered as Zn (1035 μg) > Pb (397 μg) > Cu (373 μg) > Ni (118 μg) > Cd (9.2 μg), and Zn, Ni, Pb, and Cu were significantly greater (*p* < 0.05) than the corresponding PEA in the PT by 307%, 276%, 235%, 279%, 233%, and 191%, respectively (Figure 7d). The extraction capacity of ornamental plants is much lower than that of super-enriched plants. For example, the extracted Cd by *R. pulchrum* was 11% and 29% of the total extracted Cd by *Solanum nigrum* L., which were 212 and 81.5 μg Cd with and without IAA application, respectively [34]. Usually, hyperaccumulators can extract 100 times more heavy metals than nonhyperaccumulators [62], but the advantage of ornamental plants is that they have ornamental value, which can be used for urban greening, and more importantly, they have a long and continuous remediation time, which can make up for the defect of small extraction ability.

The applying IAA did not significantly change the PEA of heavy metals of *R. pulchrum*, but the dose of 20 ppm IAA significantly decreased (*p* < 0.05) the PEA of heavy metals in *L. rubrum* by 19–59% compared to CK. This result was consistent with the previous results, such as the unchanged biomass and heavy metal concentrations in plants in most cases after the application of IAA. The results also demonstrated that the effect of IAA on the accumulation of heavy metals in plants depends on the plant species, the concentration of spraying IAA and other factors [31]. Therefore, the dose and frequency of phytoregulators might need to be increased in the dissipation environment in the open field.

### 3.7. Correlation Analysis

The result of the correlation analysis showed that the soil physicochemical properties, such as soil pH, SOM, and CEC, had little impact on the migration of heavy metals in the ornamental plants in most cases (Tables S1 and S2). There were significant correlations between plant growth index and BCF values of heavy metals in *R. pulchrum*, especially the root biomass was significantly positively correlated (*p* < 0.05) with BCF values of all tested heavy metals except Cu, but the correlations were poor in most cases in *L. rubrum.* Usually, the physicochemical properties of soil have good relationships with the migration factors of heavy metals, such as BCF in ryegrass [32]. The difference was probably related to the two ornamental plant species selected in this study. Both plants are shrubs with tap root systems with small root density and biomass, which are difficult to make full action with the soil. In the study, the root-shoot biomass ratio (RSR) of *L. rubrum* and *R. pulchrum* was 0.42–0.52 and 0.26–0.35 (Figure S1), respectively, which were similar to the RSR 0.344 of an evergreen shrub, but smaller than the RSR 0.637 of evergreen grass reported by Qi [63]. However, the root system of *R. pulchrum* grew better than that of *L. rubrum* after transplantation (Figure 2a), which could explain *R. pulchrum* performed better than *L. rubrum* in the correlation analysis of BCF to plant growth index. 

In addition, the correlations among most BCFs and TFs of heavy metals in *R. pulchrum* were positively or negatively significant (*p* < 0.05), which verified the complex interaction of these heavy metals, including synergistic and antagonistic effects, as reference mentioned [28]. Therefore, it was deduced that the impact of the interaction of heavy metals originating from the plant itself on the uptake of heavy metals was stronger than that from the external factors, such as applied exogenous phytohormone. In addition, under heavy metal stress, the synthesis of stress-related hormones and antioxidants could also be initiated in plants, as reported [64], which could mask the effect of exogenous phytohormone. Therefore, it could be explained that the application of plant hormones IAA did not significantly improve the effect of plants on heavy metal extraction in the research.

## 4. Conclusions

In this study, the feasibility of using two garden plants as phytoremediation plants to rehabilitate heavy-metal-contaminated sewage sludge in field experiments was tested, and the role of exogenous phytohormone IAA was also examined. The two ornamental plants could adapt to the artificial soil made of municipal sewage sludge. The concentration of Pb in all parts of the two ornamental plants was increased, as well as most heavy metals in *L. rubrum* root. As a result, three months after transplant, the phyto-extraction amounts were increased by 143% to 193% for Zn, Pb, and Cu in *R. pulchrum* and were increased by 233% to 279% for Ni, Cd, and Pb in *L. rubrum*. All these results indicated a potential to remediate heavy metals of *L. rubrum* and *R. pulchrum,* especially *L. rubrum*. The results of correlation analysis implied that the interaction of heavy metals in the plant itself played an important role in the uptake of heavy metals. The effect of applying IAA on plant growth and phytoremediation on heavy metals was masked due to various causes. Therefore, there is a need to modify IAA concentrations or develop other more effective methods to assist garden plants in extracting more heavy metals from the artificial soil need to be developed. In addition, reasonable management of the biomass produced by the remediation process is also very important, for example, taking the biomass of ornamental plants out of the park (soil) to avoid secondary pollution. Therefore, the promotion of phytoremediation technology also needs to be supported by policy and law research.

## Figures and Tables

**Table 1:** The physical and chemical properties of the artificial soil made of sewage sludge.

Physicochemical Property	Soil Planted with *L. rubrum*	Soil Planted with *R. pulchrum*
pH (soil:water = 1:2.5, *n* = 9)	7.92 ± 0.09	8.05 ± 0.18
Total nutrients (%, N + P_2_O_5_ + K_2_O)	3.0 ± 1.8	3.1 ± 1.5
Soil organic matter concentration (g kg^−1^, *n* = 9)	65.0 ± 7.0	65.8 ± 8.9
Cation exchange capacity (cmol kg^−1^, *n* = 9)	110.7 ± 2.3	110 ± 1.3
Cu concentration (mg kg^−1^, *n* = 9)	71.0 ± 2.1	68.6 ± 1.5
Zn concentration (mg kg^−1^, *n* = 9)	1470 ± 79	1350 ± 70
Pb concentration (mg kg^−1^, *n* = 9)	137 ± 11	114.7 ± 9.7
Cd concentration (mg kg^−1^, *n* = 9)	6.6 ± 3.0	6.9 ± 2.2
Ni concentration (mg kg^−1^, *n* = 9)	115.8 ± 9.0	105.3 ± 5.9

## Data Availability

No applicable.

## Supplementary Materials

The following supporting information can be downloaded at: https://www.mdpi.com/article/10.3390/toxics11010043/s1, Table S1: Pearson correlation coefficients of migration factor of heavy metals with physicochemical properties of soil and *R. pulchrum*.; Table S2: Pearson correlation coefficients of migration factors of heavy metals with physicochemical properties of soil and *L. rubrum*. Figure S1: Root-shoot biomass ratio of *L. rubrum* and *R. pulchrum* before and after IAA treatments.
