# Supplementary material for: Utilization of Indole Acetic Acid with Leucadendron rubrum and Rhododendron pulchrum for the Phytoremediation of Heavy Metals in the Artificial Soil Made of Municipal Sewage Sludge
Source: Toxics. 2022 Dec 31;11(1):43. doi: 10.3390/toxics11010043 (PMC9864706; doi:10.3390/toxics11010043)

Table S1

Pearson correlation coefficients of migration factor of heavy metals with physicochemical properties of soil and *R. pulchrum*.

Item	pH	SOM	CEC	Chlorophyll	Leaf Biomass	Stem Biomass	Root Biomass	BCF-Cu	BCF-Zn	BCF-Pb	BCF-Cd	BCF-Ni	TF-Cu	TF-Zn	TF-Pb	TF-Cd	TF-Ni
pH, *n*=36	1	○0.289	🔴-0.837	○0.213	○0.235	○-0.292	●-0.384	●0.403	○0.327	○-0.076	○-0.066	○0.082	○0.268	○-0.286	○0.279	○0.205	🔴-0.552
SOM, *n*=36		1	●-0.369	○0.048	○0.068	○-0.056	○-0.210	○-0.144	○-0.302	●-0.412	○-0.330	○-0.085	○0.082	○0.057	○0.136	○-0.065	○-0.145
CEC, *n*=36			1	○-0.258	○-0.172	○0.082	○0.243	●-0.392	●-0.432	○0.169	○0.103	○0.089	○-0.171	●0.373	○-0.142	○-0.120	●0.401
Chlorophyll, *n*=36				1	○-0.124	○-0.172	●-0.373	○0.179	○-0.116	●-0.426	●-0.384	○-0.313	○0.125	○0.021	○0.243	○0.234	○0.159
Leaf Biomass, *n*=36					1	🔴0.451	🔴0.429	○0.043	●0.414	●0.407	○0.328	●0.448	○-0.271	○-0.325	○-0.134	○-0.178	○-0.256
Stem Biomass, *n*=36						1	🔴0.730	○-0.242	○0.076	○-0.060	○-0.048	○-0.103	○-0.039	○0.217	○-0.028	○-0.197	🔴0.451
Root Biomass, *n*=36							1	○0.205	●0.477	●0.374	●0.451	●0.382	🔴-0.428	○-0.136	○-0.307	🔴-0.511	○0.222
BCF-Cu, *n*=27								1	●0.435	○0.154	○0.279	●0.440	●-0.434	🔴-0.602	○-0.305	●-0.422	🔴-0.538
BCF-Zn, *n*=27									1	🔴0.669	🔴0.554	●0.457	🔴-0.790	🔴-0.921	🔴-0.730	🔴-0.603	●-0.427
BCF-Pb, *n*=27										1	🔴0.832	🔴0.694	🔴-0.703	🔴-0.667	🔴-0.786	●-0.457	●-0.439
BCF-Cd, *n*=27											1	🔴0.794	🔴-0.631	🔴-0.603	🔴-0.581	🔴-0.627	🔴-0.559
BCF-Ni, *n*=27												1	🔴-0.742	🔴-0.593	🔴-0.648	🔴-0.631	🔴-0.782
TF-Cu, *n*=36													1	🔴0.700	🔴0.801	🔴0.586	○0.137
TF-Zn, *n*=36														1	🔴0.586	●0.368	🔴0.578
TF-Pb, *n*=36															1	○0.304	○0.084
TF-Cd, *n*=36																1	○0.091
TF-Ni, *n*=36																	1

●, 🔴 means significant at $p < 0.05$ and $p < 0.01$ (two-tailed), respectively; ○ means not significant.

Table S2

Pearson correlation coefficients of migration factors of heavy metals with physicochemical properties of soil and *L. rubrum*.

Item	pH	SOM	CEC	Chlorophyll	Leaf Biomass	Stem Biomass	Root Biomass	BCF-Cu	BCF-Zn	BCF-Pb	BCF-Cd	BCF-Ni	TF-Cu	TF-Zn	TF-Pb	TF-Cd	TF-Ni
pH, *n*=36	1	-0.079	○-0.021	○0.092	○-0.154	○0.039	○-0.204	○-0.065	○0.024	○-0.120	○-0.299	○-0.223	○-0.163	○-0.095	○0.185	○0.234	🔴0.438
SOM, *n*=36		1	○-0.236	○-0.069	🟢0.356	○-0.220	🟢0.360	○0.380	🟢0.457	○0.142	○0.233	○0.164	○-0.016	🟢-0.348	○-0.105	🟢-0.359	○-0.176
CEC, *n*=36			1	○0.028	🟢0.423	○0.057	○0.072	🔴-0.845	🔴-0.908	○-0.191	○0.033	🟢-0.413	🟢-0.344	○-0.157	🔴-0.559	🟢0.378	○-0.197
Chlorophyll, *n*=36				1	○0.033	○0.173	○0.057	🔴0.495	🟢0.442	🟢0.470	○-0.085	🟢0.448	🔴-0.492	🟢-0.386	○-0.110	○0.279	○0.031
Leaf Biomass, *n*=36					1	○0.215	🔴0.719	○0.118	○0.093	○0.241	○0.117	○0.178	🟢-0.336	🔴-0.613	🔴-0.580	○0.025	🔴-0.455
Stem Biomass, *n*=36						1	🟢0.360	○0.053	○0.023	○0.239	○0.275	🔴0.521	○-0.018	○-0.009	○0.247	○-0.129	○-0.072
Root Biomass, *n*=36							1	○0.225	○0.296	○0.301	○0.183	○0.338	○-0.101	🟢-0.417	○-0.292	○-0.013	🔴-0.467
BCF-Cu, *n*=27								1	🔴0.951	🟢0.476	○0.076	🔴0.562	🔴-0.874	🔴-0.751	○0.066	🟢-0.402	○0.069
BCF-Zn, *n*=27									1	🟢0.434	○0.042	🔴0.526	🔴-0.752	🔴-0.783	○0.074	○-0.321	○-0.033
BCF-Pb, *n*=27										1	○0.224	🔴0.683	○-0.262	○-0.326	○0.064	○-0.228	○-0.366
BCF-Cd, *n*=27											1	○0.330	○0.127	○-0.049	○0.174	🔴-0.506	○-0.054
BCF-Ni, *n*=27												1	○-0.363	🟢-0.440	○0.323	🟢-0.434	○-0.340
TF-Cu, *n*=36													1	🔴0.841	🔴0.581	○-0.071	○-0.023
TF-Zn, *n*=36														1	🔴0.637	○0.014	○0.235
TF-Pb, *n*=36															1	○-0.280	🟢0.419
TF-Cd, *n*=36																1	○-0.051
TF-Ni, *n*=36																	1

🟢, 🔴 means significant at $p < 0.05$ and 0.01(two-tailed), respectively; ○ means not significant.

Figure S1. Root-shoot biomass ratio of *L. rubrum* and *R. pulchrum* before and after IAA treatments. The letter in the same column of the same plant indicate significant differences among the treatments, $p < 0.05$.

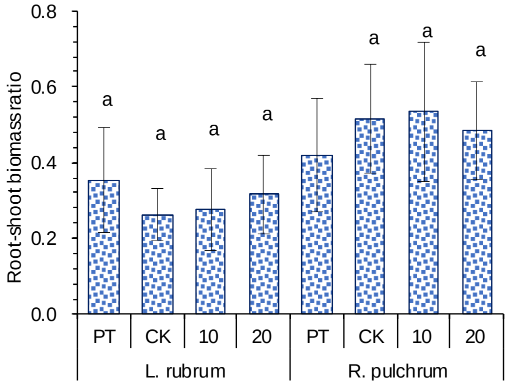

Supplement: Supplementary file 1 [file toxics-11-00043-s001.zip › toxics-2097383-supplementary.pdf]
